# Musculoskeletal Attendances to a Minor Injury Department During a Pandemic

**DOI:** 10.7759/cureus.13143

**Published:** 2021-02-05

**Authors:** Richard J Dowell, Neil Ashwood, Jamie Hind

**Affiliations:** 1 Emergency Department, University Hospitals of Derby and Burton, Derby, GBR; 2 Trauma and Orthopaedics, University Hospitals of Derby and Burton, Derby, GBR; 3 Trauma and Orthopaedics, Walsall Manor Hospital, Walsall, GBR

**Keywords:** musculoskeletal injuries, msk injuries, minor injuries, fractures, emergency department attendances, covid 19

## Abstract

Introduction: As coronavirus disease 2019 (COVID-19) became a public health emergency of international concern, countries across the globe began to instate strict social distancing restrictions or “lockdowns”. During these times emergency departments in the United Kingdom (UK) recorded a significant drop in patients attending when compared to the same months of previous years. Attendances related to musculoskeletal (MSK) trauma also saw a significant drop in numbers

Objective: The purpose of this retrospective audit was to investigate patterns of injuries attending during the pandemic and more specifically during times of lockdown.

Method: Retrospective audit data was collected from an electronic medical record system (MediTech V6) during the time period of the first lockdown in the UK. Data was collected for patients attending the emergency department at the Queens Hospital Burton site of the University Hospitals of Derby and Burton National Health Service (NHS) Trust. Presenting complaints were recorded for the entire emergency department, and diagnosis on discharge and activity status was recorded for minor injuries only. This data was then compared to the same date from 2019.

Results: Overall attendances in the emergency department decreased by 45.42% during the first lockdown when compared to the same time period in 2019. MSK problems also saw a significant drop as back pain decreased by 58.88%, neck pain fell by 78.52% and limb problems decreased by 59.74%. When comparing data from the minor injury department, limb problems decreased by 20.45%. The number of soft tissue injuries decreased by 24.05% and fractures decreased by 7.96%.

Conclusion: Attendances in the emergency department were greatly reduced during the COVID-19 pandemic, especially during the first lockdown. The rates of fractures and soft tissue injuries within the minors’ area of the emergency department were also reduced but not at the same rate as the overall attendance. A large number of fractures and soft tissue injuries still presented to the emergency department despite reduced national activity. These attendances may be as a result of the increased rate of Do It Yourself (DIY)-related injuries and altered patient/social behaviour due to lockdown, social distancing, and seasons/weather. Further research would be required to investigate the changing patterns of behaviour especially as we enter a second wave of cases.

## Introduction

In December 2019, in Wuhan, Hubei province, China, the first case of coronavirus disease 2019 (COVID-19) was reported [1]. By January 31, COVID-19 had spread to 19 countries [2] and was declared a public health emergency of international concern by the World Health Organisation (WHO) [3]. The first cases of COVID-19 diagnosed in the United Kingdom (UK) were made the week commencing January 27, 2020 [4]. At this time the virus had spread significantly with over 7,000 cases in China and 90 cases elsewhere [5]. Evidence suggested that the mode of transmission of COVID-19 was human to human [6], with the major route of transmission via respiratory droplets and close human contact [7]. It was declared a global pandemic by the WHO on March 11, 2020 [8]. On March 12 the UK government announced it was moving from the containment phase to the delay phase, preparing the public for the introduction of strict measures of social distancing [9]. By March 23, 2020, COVID-19 claimed 335 lives in the UK and it was at this time when the UK went into "lockdown" whereby strict guidelines were introduced to minimise the spread of the virus [10]. These guidelines promoted social distancing by encouraging the general public to stay indoors, only leaving for essential shopping, medical needs, and exercise once a day. This meant many people were encouraged to work from home or placed on the Furlough program where workers were paid 80% of salary whilst staying at home and not working. It was reported 6.3 million UK workers were placed on the Furlough program in early May 2020 [11]. On March 20 schools were closed in the UK to the majority of pupils, allowing only the children of healthcare workers to attend [12]. Children of school age were expected to follow the strict guidelines to reduce the transmission rate of the virus.

During the time of strict social distancing guidelines, attendances in the National Health Service (NHS) Emergency Departments decreased by 43.5% when compared to attendances for the same months in 2019. In 2020 attendances from March 1 to May 31 totalled 6,451,722 across all Emergency Departments and minor injury units across England, attendances decreased to 3,710,218 between the same dates in 2020 [13]. The greatest fall of attendances was during the month of April 2020 whereby the number of people using Emergency Departments decreased to below one million (916,581). 

The purpose of this study was to use retrospective data taken from the emergency department to assess which musculoskeletal (MSK) conditions attended the emergency department during a pandemic when compared to the same months of the previous year.

## Materials and methods

Data was collected from Meditech Version 6, an online clinical operating system allowing electronic documentation, tracking and management of patients attending the emergency department at a single site. Diagnostic codes are used as part of the documentation process allowing data to be extracted and analysed retrospectively. The data categories used for this study were hospital number, age, presenting complaint, department (minor vs major), and discharge diagnosis.

Data was extracted between the dates of March 13 to May 13, comparing attendances in both 2019 and 2020. Presenting complaint was used to compare all patients attending the emergency department (majors and minors). Diagnosis on discharge was used when comparing the MSK conditions presenting in the emergency department. The discharge data was used as it reflects a more accurate diagnosis of the condition. Data from the majors' area of the emergency department was excluded at this stage due to a large portion of missing data.

Initial analysis was performed comparing presenting complaints for the entire emergency department attendances. As patients are booked into the emergency department and categorised based on the presenting complaint. Number of attendances for each presenting complaint category was then compared between March 13 and May 13, 2019 to 2020. The data was then divided by the two areas where the patients were seen, data collected from patients placed in the majors' area of the emergency department was excluded at this stage due to a large proportion of missing data recorded at discharge. As the focus of the study was to assess the change in patterns of MSK injuries, presenting the data from limb problems placed under the minors' area was used to compare year groups.

As a secondary investigation, the age of patients presenting in the emergency department during a pandemic compared to the previous non-pandemic year was assessed to see if injury patterns changed within this demographic meaningfully. 

## Results

Overall attendances between the dates of March 13 and May 13 in the emergency department at Queens Hospital Burton reduced from 12,608 to 6,882, a reduction of 45.42% when comparing 2019 to 2020. Musculoskeletal presentations to the emergency department also declined. Back pain reduced by 58.88% from 321 in 2019 to 132 in 2020; neck pain reduced by 78.52% from 135 in 2019 to 29 in 2020, and limb problems reduced by 59.74% from 2889 in 2019 to 1163 in 2020 (Table 1).

**Table 1:** Presenting complaints March 13 – May 13, 2019 vs 2020

Presenting Complaint	2019	2020	Change (%)
Abdominal pain	889	489	-44.99%
Abscess/infection	154	122	-20.78%
Alcohol related	49	13	-73.47%
Allergic reaction	50	29	-42.00%
Assault	36	22	-38.89%
Asthma	48	21	-56.25%
Back pain	321	132	-58.88%
Bite	58	31	-46.55%
Behaviour	5	8	60.00%
Burn	45	28	-37.78%
Chemical exposure	21	6	-71.43%
Collapse	195	122	-37.44%
Crying baby	7	2	-71.43%
D&V	98	47	-52.04%
Dental	53	18	-66.04%
Diabetic problem	61	32	-47.54%
Ear problem	76	39	-48.68%
Eye problem	340	150	-55.88%
Foreign body	133	65	-51.13%
Fits	131	68	-48.09%
Falls	245	197	-19.59%
Generally unwell	2083	1386	-33.46%
GI bleed	99	53	-46.46%
Headache	183	89	-51.37%
Fascial injury	254	87	-65.75%
Head injury	529	227	-57.09%
Limping child	29	4	-86.21%
Limb problem	2889	1163	-59.74%
Major trauma	16	13	-18.75%
Mental health	141	79	-43.97%
Neck pain	135	29	-78.52%
Overdose	169	103	-39.05%
Palpitations	128	83	-35.16%
Pregnancy	114	64	-43.86%
PV bleed	133	91	-31.58%
Rash	106	36	-66.04%
Sexual acquired infection	1	2	100.00%
Social admission	20	14	-30.00%
Shortness of breath	735	638	-13.20%
Sorethroat	98	44	-55.10%
Testicle pain	40	30	-25.00%
Torso injury	81	32	-60.49%
Urinary problem	307	189	-38.44%
Worried parent	81	21	-74.07%
Wound	216	246	-40.87%
Total	12608	6882	-45.42%

Although overall presentations of limb problems reduced by 59.74% when comparing the attendances for the entire emergency department. When comparing the attendances allocated to the minors' area, limb problems only decreased by 20.45%, from 709 in 2019 to 564 in 2020 (Table 2). Soft tissue injuries decreased by 24.05% from 395 in 2019 to 300 in 2020 and fractures decreased by 7.96% from 201 in 2019 to 185 in 2020.

**Table 2:** Discharge diagnosis of patient presenting with “Limb problems” located in Minors CES: cauda equina syndrome, DVT: deep vein thrombosis

Discharge Diagnosis	2019	2020	Change (%)
Abscess	4	0	-100.00%
Bursitis	3	4	33.33%
Cellulitis	5	4	-20.00%
CES	2	0	-100.00%
Chronic pain	3	0	-100.00%
DVT	6	1	-83.33%
Foreign Body	2	0	-100.00%
Fracture	201	185	-7.96%
Gout	2	3	50.00%
Haemarthrosis	2	0	-100.00%
Head injury	8	0	-100.00%
Infection	1	3	200.00%
Left before assessment	2	0	-100.00%
No Abnormality	14	7	-50.00%
Neurology	1	0	-100.00%
Osteoarthritis	13	7	-46.15%
Plaster Problem	6	3	-50.00%
Post-op Problem	1	0	-100.00%
Pulled Elbow	2	2	0.00%
Inflammatory Arthritis	2	2	0.00%
Sciatica	8	9	12.50%
Septic Arthritis	2	2	0.00%
Social Problem	1	0	-100.00%
Soft Tissue Injury	395	300	-24.05%
Tendinopathy	9	12	33.33%
Vascular	2	0	-100.00%
Wound	12	20	66.67%
Total	709	564	-20.45%

The percentage of the overall attendance which was classified as a limb problem decreased from 22.91% to 16.90%, wounds and fractures discharged from the minors' area increased from 3.30% to 3.57% and 1.59% to 2.69% respectively (Table 3).

**Table 3:** Percentage of the overall attendance made up of Limb problems, wounds and fractures in the emergency department and minors area.

Year	Total Attendances	Limb Problems	%	Wounds	%	Fractures located in Minors	%
2019	12,608	2,889	22.91%	416	3.30%	201	1.59%
2020	6,882	1,163	16.90%	246	3.57%	185	2.69%

The age range of patients presenting to the emergency department during a pandemic did not differ greatly when compared to non-pandemic times. The average age did increase by five years in 2020 (Table 4).

**Table 4:** Patient average and range of age presenting to the emergency department

Year	2019	2020
Age range	12days – 105years	10days – 101years
Average age	45years	50years

Activity status of MSK injuries attending the minors' area of the emergency department differed significantly during times of a pandemic. Attendances relating to gardening increased from 13 to 28, indoor recreation (such as hobbies) increased from 44 to 52, and Do It Yourself (DIY) from 37 to 52. In comparison attendances relating to road traffic collisions (RTC) reduced from 70 to eight, injuries at the gym reduced from 21 to eight, and football-related injuries reduced from 46 to 19 (Table 5). 

**Table 5:** Activity status during injury attending minor injury area of the emergency department DIY: Do It Yourself

Activity	2019	2020	% change
Gardening	13	28	+115.4%
Indoor recreation	44	52	+40.9%
DIY	37	52	+40.5%
Gym	21	8	-61.9%
Road Traffic Collision	70	8	-88.6%
Football	46	19	-58.7%
Walking	336	184	-45.2%
Social: Play/Recreation	121	73	-39.7%
Labour	37	23	-37.8%

**Figure 1:**
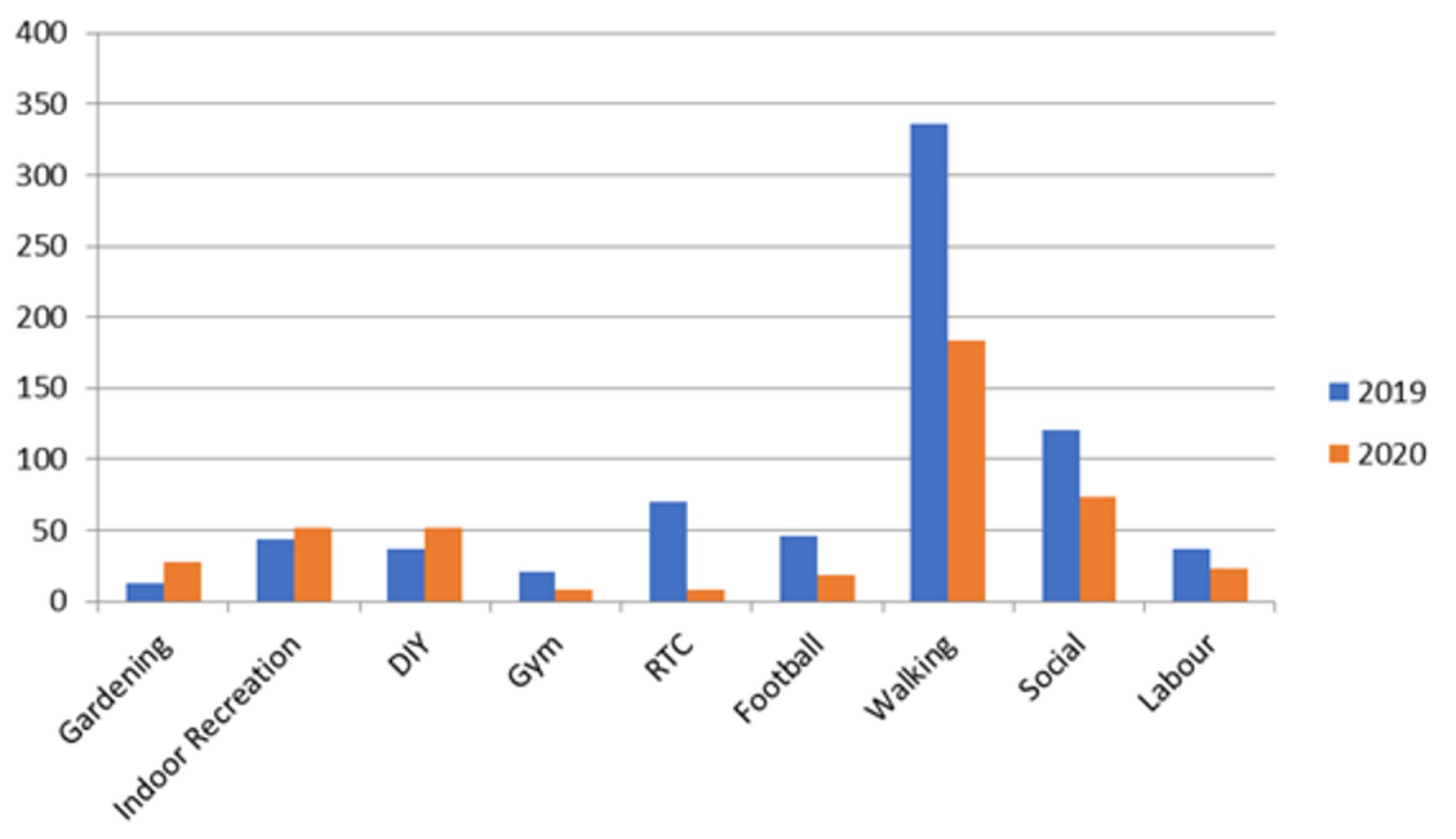
Activity status of musculoskeletal injuries presenting in minors area in the emergency department DIY: Do It Yourself, RTC: road traffic collisions

The type of activities resulting in injuries and therefore attendance to the emergency department differed during the pandemic. Certain activities such as DIY housework, indoor recreations, and gardening increased during the pandemic whereas activities such as sports, driving, and work decreased, as shown in Figure 1.

**Figure 2:**
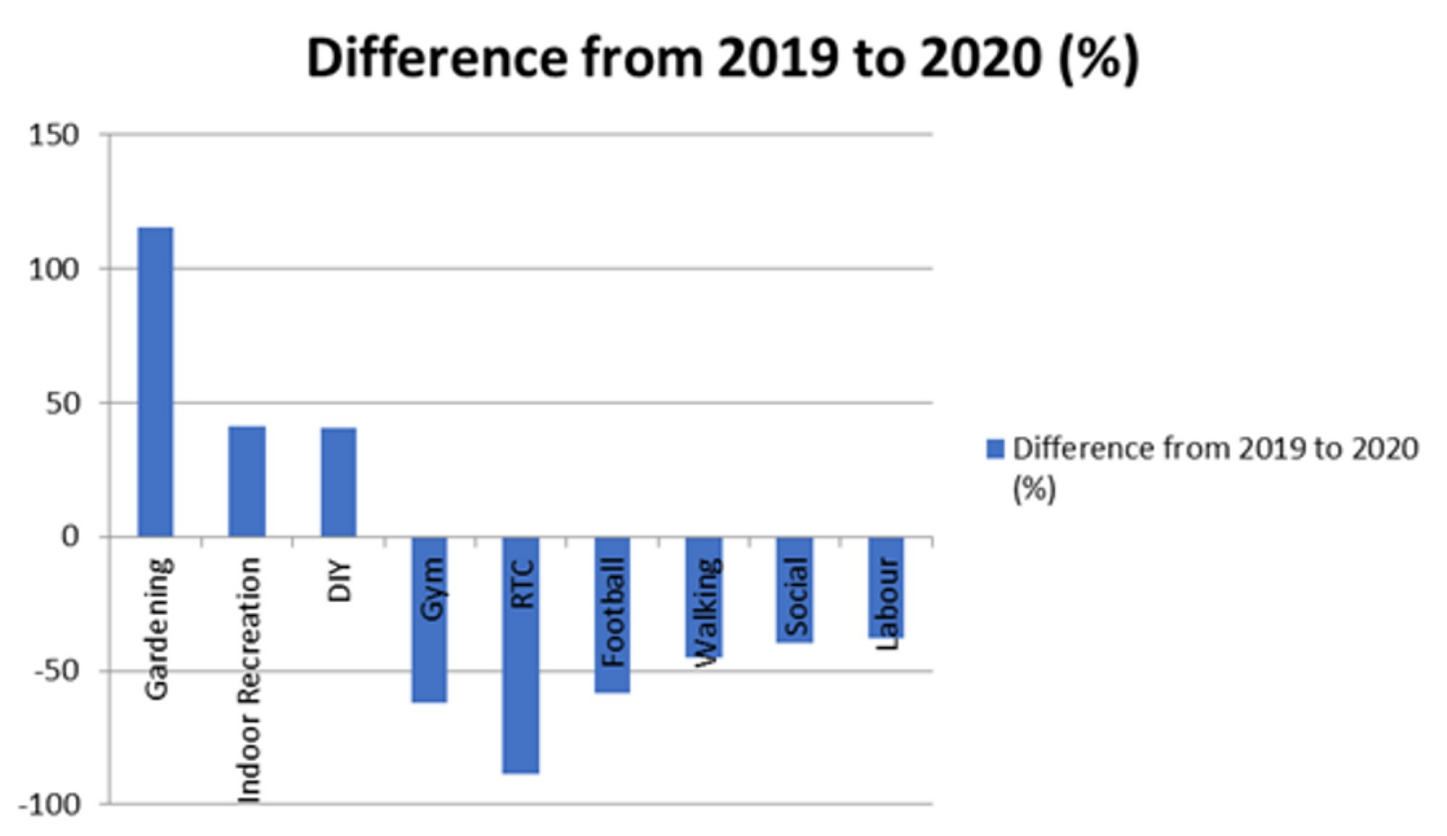
Activity status difference between 2019 and 2020. DIY: Do It Yourself, RTC: road traffic collisions

Figure 2 shows the difference in activity status attending during times of a pandemic in comparison to non-pandemic times. Gardening-related injuries increased by 115.4%, indoor recreation-related injuries increased by 40.9% and DIY increased by 40.5%. In comparison, RTC reduced by 88.6%, gym-related injuries reduced by 61.9%, and football-related injuries reduced by 58.7%. 

## Discussion

On March 23 2020 the United Kingdom government introduced six weeks of strict social distancing guidelines in response to the COVID-19 global epidemic. From March 1 to May 31, 2020, attendances in the emergency department decreased by 43.50% across England in comparison to the same months in 2019. The greatest decline in attendances was seen during the month of April 2020, the middle of strict social distancing guidelines. Emergency department attendances at the Queens Hospitals in Burton on Trent decreased by 45.42%, consistent with the average drop in attendances seen in emergency departments in England. The reduction in attendances is likely to be as a result of the strict social distancing guidelines. Consequently bars, restaurants and gyms were forced to close, contact sports stopped and many workers across the United Kingdom were either placed on a furlough scheme or encouraged to work from home. The reduced activity across the United Kingdom is likely to be a direct cause of the reduction in illness/injury and therefore reduction in attendees at the emergency department. Attendances may also be reduced due to much of the media coverage during this time stressing the importance of social distancing and the need to ease pressure on the National Health Service. This may have discouraged people from attending the emergency department partly due to fear of coming into contact with a contagious virus and partly due to the hope of easing pressures on the health services. Although overall emergency department attendances decreased by 45.42% at Queens Hospital Burton, the number of fractures seen in the minors' area of the department only decreased by 7.96% from 201 (2019) to 185 (2020). Despite the large decline in patients coming to the emergency department and the strict social distancing guidelines there was still a significant number of fractures seen in the minors' area. Soft tissue injuries were also reduced from 395 (2019) to 300 (2020), a reduction of 24.05%. There were still a large number of soft tissue injuries present in the emergency department during the period of reduced attendances, and like with the fracture this may in part be explained by an increase in DIY, gardening, and indoor recreation-related injuries. Although work and sport-related injuries decreased during the period of strict social distancing, DIY-related injuries increased. Labour related injuries fell by 37.8% during the period of social distancing when compared to 2019, and gym-related injuries (-61.9%), driving-related injuries (-88.6%), and football-related injuries (-58.7%) also saw a significant decrease during the strict social distancing, as shown in Figure 1 and Figure 2. The large decrease in these activities is explained by the population being encouraged to stay at home, this may also explain the increase in gardening (115.4%), DIY (40.5%) and indoor recreational activity (40.9%)-related injuries. There was a large reduction in soft tissue injuries presenting during the strict social distancing guidance in comparison to fractures (-24.05% for soft tissue injuries vs -7.96% for fractures). This could indicate patients adopting a higher threshold to attending the emergency department with an injury and therefore more likely to try to self-manage at home. This could increase the rate of fractures or significant injuries seen within the emergency department but further research would be required to confirm this. The increased rate of fractures seen within the minors’ area of the emergency department is reflected in the percentage of the overall attendance which increased from 1.59% in 2019 to 2.69% in 2020.

There have been numerous articles analysing trauma patterns during the COVID-19 outbreak with the pattern of injury changing depending on the country, region and age of the patients [14-15]. During the COVID-19 pandemic emergency departments internationally saw a reported reduction in patients attending during relative periods of lockdown in each country [16-24]. Overall attendances to emergency departments were reported to have declined by as much as 68% in Italy [18-19] and 60% in parts of the United States of America [20]. An article from Finland reported that although overall attendances reduced by 17%, attendance related to MSK pain or injury reduced by 31% [21]. In places such as Barcelona (Spain), trauma attendances reduced by as much as 86.1% [22]. Italy also reported a large reduction in the number of attendances related to trauma, reducing by 70%. This fall in trauma attendances being partially explained by the low numbers of sport (-96.2%) and road traffic collision (RTC) (-79.6%) related injuries [19]. Reported data seen in this audit demonstrated similarities to the reduction in RTC-related injuries declining by 88.6% however injuries related to sports such as football only reduced by 58.7%. A 52.1% decline in weekly trauma attendances were also reported in France during the period of lockdown [23]. This is consistent with the data seen in this audit with “Limb Problems” which declined by 59.74% during the period of lockdown. One other explanation offered for the drop in trauma-related attendances is the significant decline in the number of patients under the age of 17 attending with injuries which decreased by 62% as reported in an article from Canada [24]. Environmental changes in temperature and season all impact on the trauma pattern with less patients presenting in the winter normally. Viral illnesses being worse at this time of year (winter) in intensity and number of cases then the impact may be less on musculoskeletal trauma services because patients behaviours are already different at this time of year [25]. There is a fear injuries may be missed as patients fear to attend but this does not seem to be born out locally and each region/hospital should monitor its patient flow closely to husband resources and deploy them to support the pandemic response [26].

Limitations

This study is a retrospective review of the audit data recorded during the completion of electronic documentation of patients attending the emergency department. Data from the majors’ area of the emergency department is limited due to the accuracy in which the audit section of the electronic documentation is completed.

## Conclusions

Attendances in the emergency department were greatly reduced during the COVID-19 pandemic, especially during the first lockdown. The rates of fractures and soft tissue injuries within the minors’ area of the emergency department were also reduced but not at the same rate as the overall attendance. A large number of fractures and soft tissue injuries still presented to the emergency department despite reduced national activity. These attendances may be as a result of the increased rate of DIY-related injuries and altered patient/social behaviour due to lockdown, social distancing, and seasons/weather. Further research would be required to investigate the changing patterns of behaviour especially as we enter a second wave of cases.
